# Maternal and Parent-of-Origin Gene–Environment Effects on the Etiology of Orofacial Clefting

**DOI:** 10.3390/genes16020195

**Published:** 2025-02-04

**Authors:** Nikola Rasevic, Joseph Bastasic, Michele Rubini, Mohan R. Rakesh, Kelly M. Burkett, Debashree Ray, Peter A. Mossey, Borut Peterlin, Mohammad Faisal J. Khan, Amin Ravaei, Luca Autelitano, Maria C. Meazzini, Julian Little, Marie-Hélène Roy-Gagnon

**Affiliations:** 1 School of Epidemiology and Public Health, University of Ottawa, 600 Peter Morand Crescent, Ottawa, ON K1G 5Z3, Canada; nikolarasevic1234@gmail.com (N.R.); jbast019@uottawa.ca (J.B.); mrakesh@uottawa.ca (M.R.R.); jlittle@uottawa.ca (J.L.); 2 Department of Neuroscience and Rehabilitation, University of Ferrara, Via Ludovico Ariosto 35, 44121 Ferrara, Italy; rub@unife.it (M.R.); khnmmm@unife.it (M.F.J.K.); rvamna@unife.it (A.R.); 3 Department of Mathematics and Statistics, University of Ottawa, 150 Louis Pasteur Private, Ottawa, ON K1N 6N5, Canada; kburkett@uottawa.ca; 4 Department of Epidemiology, Bloomberg School of Public Health, Johns Hopkins University, 615 N. Wolfe Street, Baltimore, MD 21205, USA; dray@jhu.edu; 5 Department of Biostatistics, Bloomberg School of Public Health, Johns Hopkins University, Baltimore, MD 21205, USA; 6 World Health Organization–Collaborating Centre for Oral and Craniofacial Research, Dental Hospital and School, University of Dundee, Nethergate, Dundee DD1 4HN, Scotland, UK; p.a.mossey@dundee.ac.uk; 7 Clinical Institute of Genomic Medicine, University Medical Center, Zaloška cesta 7, 1000 Ljubljana, Slovenia; borut.peterlin@kclj.si; 8 Smile House Milan, Regional Centre for Orofacial Clefts and Craniofacial Anomalies, Department of Cranio-Maxillo-Facial Surgery, San Paolo Hospital, University of Milan, Via Festa del Perdono 7, 20122 Milan, Italy; luca.aute@gmail.com (L.A.); cmeazzini@yahoo.it (M.C.M.)

**Keywords:** orofacial clefts, parent-of-origin genetic effects, maternal genetic effects, gene–environment interaction, case-parent triads

## Abstract

**Background/Objectives:** We investigated maternal and parent-of-origin (PoO) gene-environment interaction effects on the risk of nonsyndromic orofacial clefts for two maternal environmental factors: periconceptional smoking and folic acid supplementation. **Methods:** Genome-wide single nucleotide polymorphisms (SNPs) genotypes and TopMed-imputed genotypes were obtained for case-parent triads from the EUROCRAN and ITALCLEFT studies. Candidate regions were selected around target SNPs from a previous genome-wide association study, resulting in 12 (726 SNPs) and 11 regions (730 SNPs) for maternal and PoO effects, respectively. Log-linear models were used to analyze 404 case-parent triads and 40 case-parent dyads. *p*-values were combined across regions. **Results:** None of the interactions reached statistical significance after correction for the number of regions tested. Nominally significant (pooled *p*-values < 0.05) interactions pointed to regions in or close to genes *LRRC7* (maternal gene-folate interaction), *NCKAP5* (PoO-smoking interaction), and *IFT43* and *GPATCH2L* (PoO-folate interaction). **Conclusions:** Our results suggested that the genetic effects in or around these genes were heightened under periconceptional exposure to tobacco or no folic acid supplementation. The involvement of these genes in orofacial cleft development, in conjunction with environmental exposures, should be further studied.

## 1. Background

Two distinct subtypes of orofacial clefts (OFCs) congenital anomalies are recognized: cleft lip with or without cleft palate (CL/P) and cleft palate only (CPO) [1]. The prevalence of OFC among live births in Europe is estimated to be 1.55 per 1000 [2]. The mental and physical health of children with OFCs are impacted in several ways, including psychology, cognition, speech, hearing and appearance. Children and teenagers with OFCs require care from a multidisciplinary team of health professionals and have been reported to have elevated morbidity and mortality rates [3,4].

OFCs have a complex etiology affected by both genetic and environmental risk factors. A cohort study based on Norwegian birth registry data indicated that the recurrence risk of OFCs is high, hinting that OFCs have genetic risk factors [5]. Genetic mechanisms that could have an influence on OFCs are maternal genetic effects and parent-of-origin (PoO) genetic effects. A mother’s genotype can affect her child’s phenotype by directly influencing the intra-uterine environment in which the offspring develops, referred to as maternal genetic effects [6]. This may cause the offspring to be at greater risk of a congenital anomaly if a risk allele is present in the mother’s genotype. PoO genetic effects occur when the phenotype associated with a specific allele of an offspring’s genotype is dependent on whether the allele was transmitted by the mother or the father [7]. The biological mechanism underlying PoO is genomic imprinting. The allele in a child’s genotype could be chemically marked depending on whether the mother or father passed down the allele, which would affect expression.

Shi et al. [8] performed a genome-wide association study (GWAS) to identify risk loci in over 2000 nonsyndromic OFC case-parent triads. This study considered maternal genetic effects as well as PoO genetic effects. No loci passed the genome-wide level of significance. This is consistent with previous studies indicating that the maternal genotype may not have a relevant impact on the risk of a child having OFCs [5,9]. Garg et al. [10] reanalyzed the same data with different methods in a genome-wide search for PoO effects, adding a replication sample for their most significant signals. The replication sample used by Garg et al. included participants from the EUROCRAN/ITALCLEFT studies also used in this study. The Garg et al. analysis yielded the same overall conclusion of no genome-wide significant PoO effect as was found by Shi et al. However, it is possible that, when ignoring environment when investigating genetic effects, statistical power is reduced if the genetic effects are modified by environmental factors.

A lower risk for OFCs was found for mother using supplements containing folic acid during early pregnancy. Mothers who took supplements containing folic acid during pregnancy had a 40% reduction in the risk of nonsyndromic CL/P and a 12% reduction in the risk of nonsyndromic CPO in a meta-analysis [11]. A positive-dose response association was observed between OFCs and maternal smoking during the first trimester [12,13]. Passive maternal smoking during pregnancy is associated with a 1.5-fold increase in the risk of a child being born with nonsyndromic OFCs [13]. To our knowledge, there is no study of the effect of interactions between maternal genetic and environmental factors on OFCs.

In this study, the most significant associations from Shi et al. (2012) were further investigated for their interaction with two environmental factors: maternal smoking and maternal use of supplements containing folic acid in populations in which folic acid fortification is not mandated. A total of 404 case-parent triads and 40 dyads from various populations in Europe in the EUROCRAN and ITALCLEFT studies were analyzed [14]. Log-linear models were applied to investigate the interaction of maternal and PoO genetic effects with maternal folic acid consumption and smoking.

## 2. Materials and Methods

### 2.1. Study Sample and Data Collection

Children with nonsyndromic OFCs and their mothers and fathers were recruited between 2001 and 2005 through the European Collaboration on Craniofacial Anomalies study (EUROCRAN; the Netherlands, United Kingdom, Spain, Hungary, Bulgaria, Estonia and Slovakia) and the ITALCLEFT study (Italy) [14]. Nonsyndromic OFCs diagnosis was confirmed at surgical centers. Infants with recognized syndromes or Pierre Robin sequence were excluded. The ITALCLEFT study, data were collected at the time of primary surgery on demographics, clinical diagnosis, pregnancy details and complications and exposure to environmental risk factors during the periconceptional period (three months before to three months after conception). The EUROCRAN questionnaire, administered personal interview when the child was brought for primary surgery, included questions on demographics, pregnancy history, and lifestyle, including exposure to environmental risk factors during the periconceptional period.

Folic-acid supplementation was defined as a mother having taken at least 0.4 mg/day of folic acid or a folic acid-containing supplements for a minimum of one month during the periconceptional period. A mother was classified as a smoker if she reported having smoked at least one cigarette/day during the periconceptional period. The main outcome considered in this analysis includes all forms of nonsyndromic OFCs combined. Both studies included the collection of peripheral blood specimens or buccal cell samples from children and their parents.

### 2.2. Statistical Analysis

We used log-linear models implemented in the Haplin package version 7.3.0 [15] in the R environment [16] to test for PoO and maternal genetic effects for each SNP. Haplin uses a Wald test to assess whether the relative risk of a maternal or PoO genetic effect significantly differs between smokers and non-smokers or between folic acid consumers and non-folic acid consumers. Models in Haplin assume Hardy–Weinberg equilibrium and random mating. For the maternal effect, relative risks were estimated using a multiplicative dose response of the minor allele. For PoO effects, relative risk ratios were estimated as the ratio of the relative risk of a maternal minor allele being transmitted to the relative risk of a paternal minor allele being transmitted. Haplin includes an expectation–maximization algorithm to handle missing genotype data on parents, allowing information from dyads to be used in the analysis [15]. We also used log-linear models to test the main maternal and PoO genetic effects ignoring the environmental factors.

In order to reduce the multiple testing burden and optimize power, we tested the significance of each candidate region by combining *p*-values within a region using two approaches: Fisher’s method ,taking LD into account using an empirically-derived null *p*-value distribution as implemented in the R package poolr version 1.1-1, and the Cauchy combination test as implemented in the CCT function from the R package STAAR [17] version 0.9.6. The region significance threshold was corrected with a Bonferroni correction for the number of regions tested. Individual SNP *p*-values and region *p*-values (pooled *p*-values) were displayed by plotting the −log_10_(*p*-values) using R. Regions where the nominally significant signals came from imputed SNPs only (i.e., the genotyped SNPs were not in LD with the imputed SNPs contributing to significance) were extended to include at least one genotyped SNP in order to further understand the LD structure. The most significant SNP in each nominally significant region was further investigated in the two OFC subtypes by stratifying the triads/dyads into the CL/P and CPO subphenotypes.

Finally, we used the LDlink tools LDexpress and LDtrait to search within 20,000 base pairs upstream and downstream of our most significant SNPs. LDexpress returns associations with gene expression in multiple tissue types based on GTEx [18] for the queried variant or variants in the specified window. LDtrait returns associations with phenotypes that are significant at the suggestive level within the GWAS catalog [19].

## 3. Results

After quality control checks (Figure S1), the analysis sample included 1292 individuals (404 triads and 40 dyads, of which 26 were case-mother dyads and 14 were case-father dyads). Characteristics of the study sample are presented in Table 1. Of the 444 offspring, 317 had CL/P, 125 had CPO, and 2 were missing OFC subtype status. Approximately half of the triads were from Italy, close to 10% were from elsewhere in Western Europe (Spain, U.K), and the remainder were from Central/Eastern Europe (Hungary, Bulgaria, Estonia and Slovakia). The overall proportion of female offspring was 39%; there was a female preponderance in the offspring with CPO (54%). The proportion of mothers who reported that they had smoked in the periconceptional period was 22.3%. Almost half of the mothers reported periconceptional use of supplements containing folic acid.

**Figure 1:**
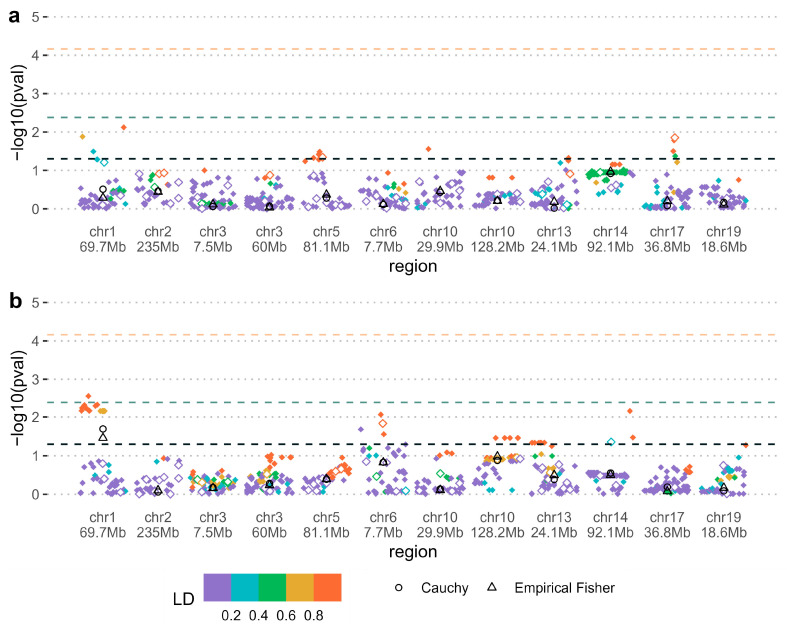
(**a**) Wald test for the interaction between maternal genetic effects and smoking; (**b**) Wald test for the interaction between maternal genetic effects and folic acid supplementation. Colors represent linkage disequilibrium (LD) structure in the region measured by r^2^ with the most significant SNP. SNP *p*-values are shown as diamond shapes, where filled shapes indicate imputed SNPs and open shapes indicate genotyped SNPs. Pooled empirical Fisher and Cauchy *p*-values are shown as open black triangles and circles. The black, green, and orange dashed lines indicate, respectively, the 0.05 significance level, the significance level for the pooled *p*-value Bonferroni-corrected for the number of regions tested, and the significance level Bonferroni corrected for the total number of SNPs tested across all regions.

**Figure 2:**
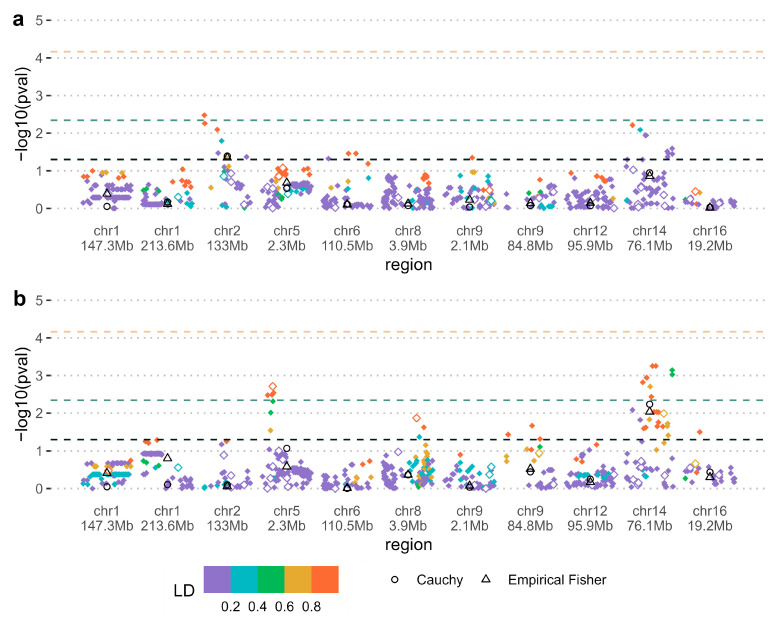
(**a**) Wald test for the interaction between parent-of-origin genetic effects and smoking; (**b**) Wald test for the interaction between parent-of-origin genetic effects and folic acid supplementation. Colors represent linkage disequilibrium (LD) structure in the region measured by r^2^ with the most significant SNP. SNP *p*-values are shown as diamond shapes, where filled shapes indicate imputed SNPs and open shapes indicate genotyped SNPs. Pooled empirical Fisher and Cauchy *p*-values are shown as open black triangles and circles. The black, green, and orange dashed lines indicate, respectively, the 0.05 significance level, the significance level for the pooled *p*-value Bonferroni corrected for the number of regions tested, and the significance level Bonferroni corrected for the total number of SNPs tested across all regions.

Figure 1 and Figure 2 present four plots of the significance of interactions of the tested genetic effects (maternal or PoO) with environmental exposure. Figure S3 shows the main maternal and PoO effects ignoring the environment. Both individual SNP interaction tests *p*-values and pooled *p*-values are shown. None of the regions reached significance after correction for the number of regions tested. One region on chromosome 5 replicated the original Shi et al. maternal effect at the nominal level before correction for the number of regions tested (Figure S3A). A region on chromosome 1 located in the *LRRC7* gene was significant at the uncorrected level for maternal gene–folate interaction, according to either Fisher’s or Cauchy’s methods (pooled *p*-values < 0.05; Figure 1b). The extended region including at least one genotyped SNP contributing to the nominally significant signal showed similar results (Figure S4A). Relative risk estimates stratified by folate supplementation status for the most significant SNP (rs12729671) in the chromosome 1 *LRRC7* region indicated a relative risk of 1.48 associated with having a copy of the minor allele when folate is not present while the relative risk was estimated to 0.73 when folate is present (Table 2). These effect estimates were consistent in triads/dyads having CL/P and in those with CPO (Table S3). 

The most significant SNP in the *LRRC7* region (rs12729671) or surrounding variants within ±20,000 base pairs were most significantly associated with gene expression in the heart and thyroid tissues (Table S4). Other phenotypes previously found to be associated with rs12729671 included chemoradiation-induced hearing loss in nasopharyngeal carcinoma (Table S5).

For PoO—smoking interactions, a region on chromosome 2 located in the *NCKAP5* gene was nominally significant according to either Fisher’s or Cauchy’s methods (pooled *p*-values < 0.05; Figure 2a). The extended region including at least one genotyped SNP contributing to the nominally significant signal showed similar results (Figure S4B). Estimates of the relative risk ratio of the maternal minor allele being transmitted vs. the paternal allele for the most significant SNP in the region were 0.60 without maternal smoking and 3.97 with maternal smoking (Table 2). These effect estimates were consistent across OFC subtypes (Table S3). The most significant SNP in the *NCKAP5* region (rs1437897) or surrounding variants within ±20,000 base pairs was associated with gene expression in the brain and testis tissues (Table S4).

An intergenic region on chromosome 14 was nominally significant for PoO—folate interaction, according to either Fisher’s or Cauchy’s methods (pooled *p*-values < 0.01; Figure 2b). Estimates of the relative risk ratio of the maternal minor allele being transmitted vs. the paternal allele for the most significant SNP in the region were 2.89 without maternal folic acid supplementation and 0.29 with maternal folic acid supplementation (Table 2). These effect estimates were consistent across OFC subtypes (Table S3). The most significant SNP in this intergenic region (rs139115930) or surrounding variants within ±20,000 base pairs was most significantly associated with gene expression in the skin, esophagus and lung tissues (Table S4). Other phenotypes previously found to be associated with rs139115930 included lung function (Table S5).

## 4. Discussion

In this study, we selected candidate regions for maternal and PoO genetic effects on the risk of OFCs with the objective of investigating their potential interaction with two environmental factors: periconceptional smoking and folic acid supplementation. None of the interactions tested reached significance after pooling *p*-values within regions and correcting for multiple testing of the number of regions with a Bonferroni correction, although some regions reached nominal significance before the Bonferroni correction. These nominally significant interactions included an interaction between maternal effects and folic acid supplementation in a region of the *LRRC7* gene on chromosome 1, an interaction between PoO effects and smoking in a region of the *NCKAP5* gene on chromosome 2, and an interaction between PoO effects and folic acid supplementation in an intergenic region on chromosome 14. 

The expression of the *LRRC7* gene is notable in the thyroid among other tissues (GTEx Release V8, dbGaP Accession phs000424.v8.p2) [18]. A recent article found a negative association between serum folate and thyroid-stimulating hormone levels in patients with type 2 diabetes mellitus [20]. Hypothyroidism (increased thyroid stimulating hormone levels and decreased thyroid hormone levels) in the mother, especially early in pregnancy, could affect the offspring’s brain development and/or lead to preterm birth, low birth weight, and respiratory distress in the neonate.

The *NCKAP5* gene is involved in microtubule bundle formation and microtubule depolymerization, as well as in the microtubule plus end [21,22]. Microtubules are involved in epithelial–mesenchymal transformation (EMT), which is a biochemical process involved in embryogenesis. In the context of OFCs, EMT on the medial edges allows for the palatal shelves to fuse [23,24]. Tobacco smoke includes many different compounds that may affect the risk of OFCs. A study illustrated that gene expressions involved with cell cycle regulation, DNA repair and oxidative stress response are affected by tobacco smoke in mice [25]. Altered DNA methylation patterns have been observed in newborns exposed to tobacco in utero [26].

The closest genes to the intergenic region on chromosome 14 are *IFT43* and *GPATCH2L*. *IFT43* is involved in intraflagellar transport. More specifically, it is involved in the intraciliary transport of particle A, intraciliary retrograde transport and the cilium itself [21,22]. It is known that ciliary dysfunction can cause OFCs [27]. *IFT43* can be related to the *NCKAP5* gene since the cilium structure is based on microtubules. On the other hand, *GPATCH2L* is involved in protein binding which may also have implications for OFC development [21,22]. The effect of alterations in the action of these genes may be heightened under folate deficiency.

Few studies have investigated effect modification by environmental factors of PoO effects [28,29,30], and results from these studies did not overlap with our findings. To our knowledge, no other study has considered the effect modification of maternal genetic effects by environmental factors. Some studies investigated interactions between offspring genotypes and in utero exposures [14,30,31,32]. We did not find common genomic regions in these studies. This is not surprising since modes of actions of maternal and PoO genetic effects in conjunction with environmental factors would likely differ or only partially overlap with the effects of the OFC child’s genome.

Among its strengths, our study has one of the largest case-parent triad sample sizes for OFCs including populations from across Europe, thus increasing the diversity of exposure. The absence of mandatory folic acid fortification programs in European countries will have resulted in lower baseline folate levels in mothers than in many other studies of OFCs, and, therefore, a ceiling effect associated with the consumption of supplements containing folic acid is unlikely. Limitations of this study include assumptions required for the analysis. When testing for associations between gene–exposure interactions in case-parent triads, independence of the child genotype and the exposure conditional on parental mating type needs to be assumed. Other assumptions include transmission ratio symmetry for the estimation of parent-of-origin genetic effects, mating symmetry for the estimation of maternal genetic effects, and the Haplin models assuming Hardy-Weinberg equilibrium. Log-linear models assuming the Hardy–Weinberg equilibrium may not have appropriate type I error control if there are differences in allele frequencies or prevalence between triads/dyads sampled from different regions of Europe [33]. However, a better alternative approach allowing tests of interaction between maternal genetic or PoO effects and environmental factors does not currently exist.

Furthermore a loss of power is expected when testing for interactions between PoO and environment since the sample is essentially split into four categories: maternal transmission with environment, maternal transmission without environment, paternal transmission with environment and paternal transmission without environment. We performed simulation-based power calculations using the hapPower function of Haplin to assess and guide the interpretation of the significance of our results (Figure S6). For maternal gene–environment interactions, we had over 80% power to detect a relative risk difference between the exposed and unexposed groups of at least 1.75 after adjusting for the multiple regions tested, while for PoO gene–environment interactions, we had ~70% power for effect sizes of 4. The suggestively significant interactions that we detected are all qualitative in the sense that the effect is reversed in the two exposure groups, thus making the effect sizes larger and easier to detect. 

Our results suggested that maternal or PoO genetic effects in or around *LRRC7*, *NCKAP5*, *IFT43,* and *GPATCH2L* were heightened under periconceptional exposure to tobacco or no folic acid supplementation. Mechanisms underlying the involvement of these genes in orofacial cleft development, in conjunction with environmental exposures, should be investigated.

## Figures and Tables

**Table 1:** Characteristics of the study sample (*n* = 444 triads/dyads).

	Number of Triads/Dyads (%) ^1^		
	All (*n* = 444)	Cleft Lip with or Without Palate (*n* = 317)	Cleft Palate Only (*n* = 125)
	Maternal periconceptual exposure		
Smoking	99 (22.3)	73 (23.0)	26 (20.8)
Supplements containing folic acid	218 (49.1)	152 (47.9)	66 (52.8)
	Child sex		
Male	272 (61.3)	213 (67.2)	57 (45.6)
Female	172 (38.7)	104 (32.8)	68 (54.4)
	Country of origin		
Italy	233 (52.5)	182 (57.4)	49 (39.2)
UK	33 (7.4)	18 (5.7)	15 (12.0)
Spain	8 (1.8)	8 (2.5)	0 (0)
Hungary	84 (18.9)	56 (17.7)	28 (22.4)
Bulgaria	31 (7.0)	25 (7.9)	6 (4.8)
Estonia	24 (5.4)	10 (3.2)	14 (11.2)
Slovakia	31 (7.0)	18 (5.7)	13 (10.4)

^1^ Data were missing for OFC subtype status (*n* = 2), smoking status (*n* = 17), and folic acid.

**Table 2:** Maternal or parent-of-origin gene–-environment interactions for the most significant SNP in nominally significant regions.

SNP	Chromosome (chr)	Minor/Other Allele (Minor Allele Frequency)	Gene	*p*-value ^1^	Pooled Region *p*-Value ^2^	Relative Risk or Relative Risk Ratio ^3^ (No Environment/Environment)
Maternal—Folic acid supplementation						
rs12729671	chr1 69.7Mb	C/T (0.22)	*LRRC7*	0.0029	0.020	1.48 [1.07, 2.08]/0.73 [0.52, 1.02]
Parent of origin—Smoking						
rs1437897	chr2 133Mb	A/G (0.26)	*NCKAP5*	0.0033	0.041	0.60 [0.33, 1.07]/3.97 [1.27, 12.03]
Parent of origin—Folic acid supplementation						
rs139115930	chr14 76.1Mb	C/T (0.15)	Intergenic	0.00056	0.0058	2.89 [1.22, 7.01]/0.29 [0.11, 0.75]

^1^ Wald test *p*-value for the interaction between maternal or parent-of-origin genetic effects and environmental factor. ^2^ Minimum of empirical Fisher’s and Cauchy’s methods. ^3^ Estimate of relative risk associated with the presence of one copy of the minor allele in the maternal genotype or of the relative risk ratio of the maternal allele being transmitted compared to the paternal allele.

## Data Availability

The data that support the findings of this study are available through Zenodo: https://doi.org/10.5281/zenodo.10928970 for researchers meeting the criteria for access. The code used to obtain the findings of this study are available on GitHub: https://github.com/Roy-Gagnon-lab/Rasevic_2024 (accessed on 24 January 2025).

## Supplementary Materials

The following supporting information can be downloaded at: https://www.mdpi.com/article/10.3390/genes16020195/s1, Table S1: Target SNPs determining candidate regions for maternal effects; Table S2: Target SNPs determining candidate regions for parent-of-origin effects; Table S3: Maternal or parent-of-origin gene–environment interaction tests in triads/dyads having a cleft palate only for the nominally significant SNPs in Table 2; Table S4: Results from LDlink/LDexpress queried for the most significant SNPs in the nominally significant regions; Table S5: Results from LDlink/LDtrait queried for the most significant SNPs in the nominally significant regions; Figure S1: Data processing steps before phasing and imputation; Figure S2: Data processing steps for phasing, imputation and afterwards; Figure S3: Plots of the maternal genetic effects and parent-of-origin effects; Figure S4: Plots of the Wald interaction test for the extended regions for nominally significant regions; Figure S5: LocusZoom plots of the three nominally significant regions; Figure S6: Estimated power to detect the maternal and PoO interaction effects.

## Abbreviations

The following abbreviations are used in this manuscript:

OFC	Orofacial cleft
CL/P	Cleft lip with or without cleft palate
CPO	Cleft palate only
SNP	Single-nucleotide polymorphism
PoO	Parent of origin
