# Supplementary material for: Maternal and Parent-of-Origin Gene–Environment Effects on the Etiology of Orofacial Clefting
Source: Genes (Basel). 2025 Feb 4;16(2):195. doi: 10.3390/genes16020195 (PMC11855025; doi:10.3390/genes16020195)
Supplement: Supplementary file 1 [file genes-16-00195-s001.zip › SupplementaryMaterial.pdf]

Supplementary Material

Summary:

Supplementary Tables S1 and S2 include the target SNPs with $p < 10^{-5}$ from Shi et al. (2012) for maternal and parent-of-origin effects, respectively. Information on the regions selected around these target SNPs is provided in the tables. SNPs that were within a region of 10,000 base pairs from the target SNPs were extracted for analysis from the imputed data.

Supplementary Table S3 includes the association results in the two OFC subtypes of the most significant SNPs in each region from the main analysis (Table 2).

Supplementary Table S4 (separate Excel file): Results from LDlink/LDexpress queried for the most significant SNPs in the nominally significant regions.

Supplementary Table S5 (separate Excel file): Results from LDlink/LDtrait queried for the most significant SNPs in the nominally significant regions.

Supplementary Figures S1 and S2 represent the data processing steps before phasing and imputation (Supplementary Figure S1) and the data processing steps for phasing, imputation and afterwards (Supplementary Figure S2).

Supplementary Figure S3: Plots of the (A) maternal genetic effects and (B) parent-of origin effects.

Supplementary Figure S4: Plots of the Wald interaction test for the extended regions for nominally significant regions (A) on chromosome 1 (chr1 69.7Mb) for maternal genetic effects and folic acid supplementation, and (B) on chromosome 2 (chr2 133Mb) for parent-of-origin effects and smoking.

Supplementary Figure S5: LocusZoom plots of the three nominally significant regions.

Supplementary Figure S6. Estimated power to detect the maternal and PoO interaction effects.

Supplementary Table S1 Target SNPs determining candidate regions for maternal effects

Target SNPs	Chromosome[a]	Target SNP position (GRCh38)[b]	Cytogenetic region	Gene located in region	Number of SNPs analyzed in region after QC
rs1417437	1	69688758	1p31.1	*LRRC7*	61
rs4505466<br>rs10174126	2	235052180<br>235052249	2q37.2	*SH3BP4*	37
rs1450100	3	7496515	3p26.1	*GRM7*	61
rs212016	3	59995799	3p14.2	*FHIT*	84
rs4703822	5	81115402	5q14.1	*RASGRF2*	42
rs2068361	6	7736510	6p24.3	*BMP6*	50
rs3006564	10	29888959	10p11.23	Intergenic	49
rs1329189	10	128200108	10q26.2	Intergenic	61
rs17079928	13	24080089	13q12.12	*SPATA13*	64
rs17807815	14	92089711	14q32.12	*ATXN3*	87
rs17138064	17	36831827	17q12	Intergenic	80
rs3764628	19	18636795	19p13.11	*KLHL26*	50

[a] Regions on chromosome 2 were overlapping and grouped for analysis.
[b] Candidate region ranged ±10kb from target SNP position.

Supplementary Table S2 Target SNPs determining candidate regions for parent-of-origin effects

Target SNPs	Chromosome	Target SNP position (GRCh38)[b]	Cytogenetic region	Gene located in region	Number of SNPs analyzed in region after QC
rs7516430	1	147295381	1q21.2	*CHD1L*	93
rs12144639	1	213643968	1q32.3	*LOC105372912*	68
rs1437903	2	133014085	2q21.2	*NCKAP5*	32
rs92833	5	2338794	5p15.33	Intergenic	132
rs11153238	6	110497644	6q21	Intergenic	51
rs1834570	8	3930208	8p23.2	*CSMD1*	111
rs10811366	9	2065892	9p24.3	*SMARCA2*	50
rs1662695	9	84790297	9q21.33	*NTRK2*	33
rs2196457	12	95880912	12q23.1	*CCDC38*	68
rs2591089	14	76121457	14q24.3	Intergenic	65
rs8054408	16	19235202	16p12.3	*SYT17*	27

[b] Candidate region ranged ±10kb from target SNP position.

Supplementary Table S3. Maternal or parent-of-origin gene-environment interaction tests in triads/dyads having cleft palate only for the nominally significant SNPs in Table 2.

SNP	Chromosome (chr)	Minor/other Allele (minor allele frequency)	Gene	P-value[a]	Region P-value[b]	Relative risk[c] (no environment / environment)
**Triads/dyads having cleft palate only**						
**Maternal - Folic acid supplementation**						
rs12729671	chr1 69.7Mb	C/T (0.22)	*LRRC7*	0.10	0.36	1.86 [0.94, 3.70] /0.86 [0.46, 1.61]
**Parent-of-origin - Smoking**						
rs58324142	chr2 133Mb	A/G (0.26)	*NCKAP5*	0.053	0.057	0.76 [0.24, 2.3] /7.73 [0.94, 59.5]
**Parent-of-origin - Folic acid supplementation**						
rs139115930	chr14 76.1Mb	C/T (0.17)	Intergenic	0.047	0.33	1.44 [0.29, 6.88] /0.14 [0.02, 0.75]
**Triads/dyads having cleft lip with or without cleft palate**						
**Maternal - Folic Acid Supplementation**						
rs12729671	chr1 69.7Mb	C/T (0.22)	*LRRC7*	0.011	0.11	1.38 [0.94, 2.02] /0.68 [0.46, 1.01]
**Parent-of-origin - Smoking**						
rs58324142	chr2 133Mb	A/G (0.26)	*NCKAP5*	0.03	0.29	0.55 [0.27, 1.09] /2.94 [0.74, 11.32]
**Parent-of-origin - Folic acid supplementation**						
rs139115930	chr14 76.1Mb	C/T (0.17)	Intergenic	0.0046	0.023	4.14 [1.43, 12.15] /0.42 [0.14, 1.34]

[a]Wald test p-value for the interaction between maternal or parent-of-origin genetic effects and environmental factor
[b]Minimum of Fisher's empirical Fisher's and Cauchy's methods.
[c]Estimate of relative risk associated with the presence of one copy of the minor allele in the maternal genotype or of the relative risk ratio of the maternal allele being transmitted compared to the paternal allele.

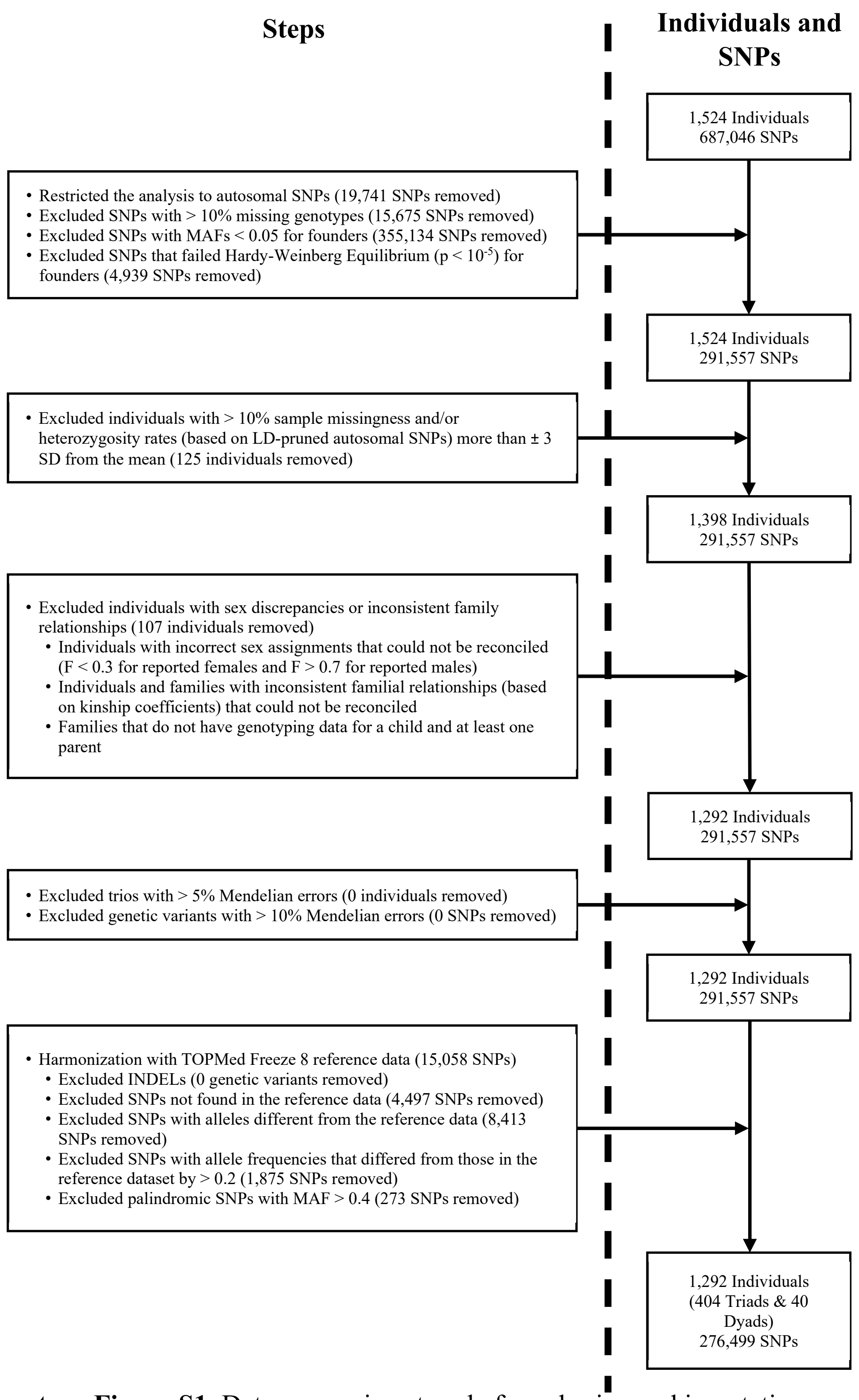

Supplementary Figure S1. Data processing steps before phasing and imputation.

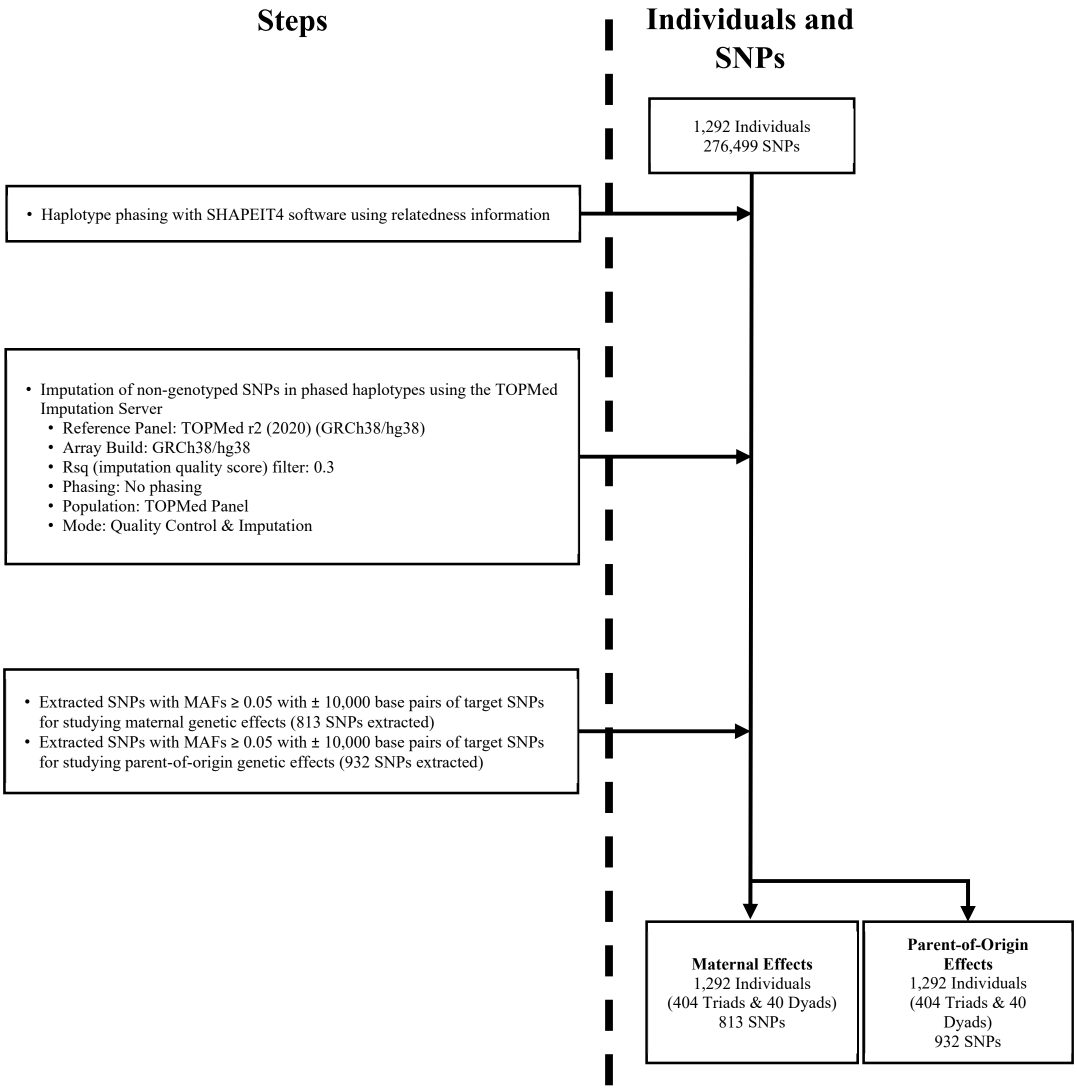

Supplementary Figure S2. Data processing steps for phasing, imputation and afterwards.

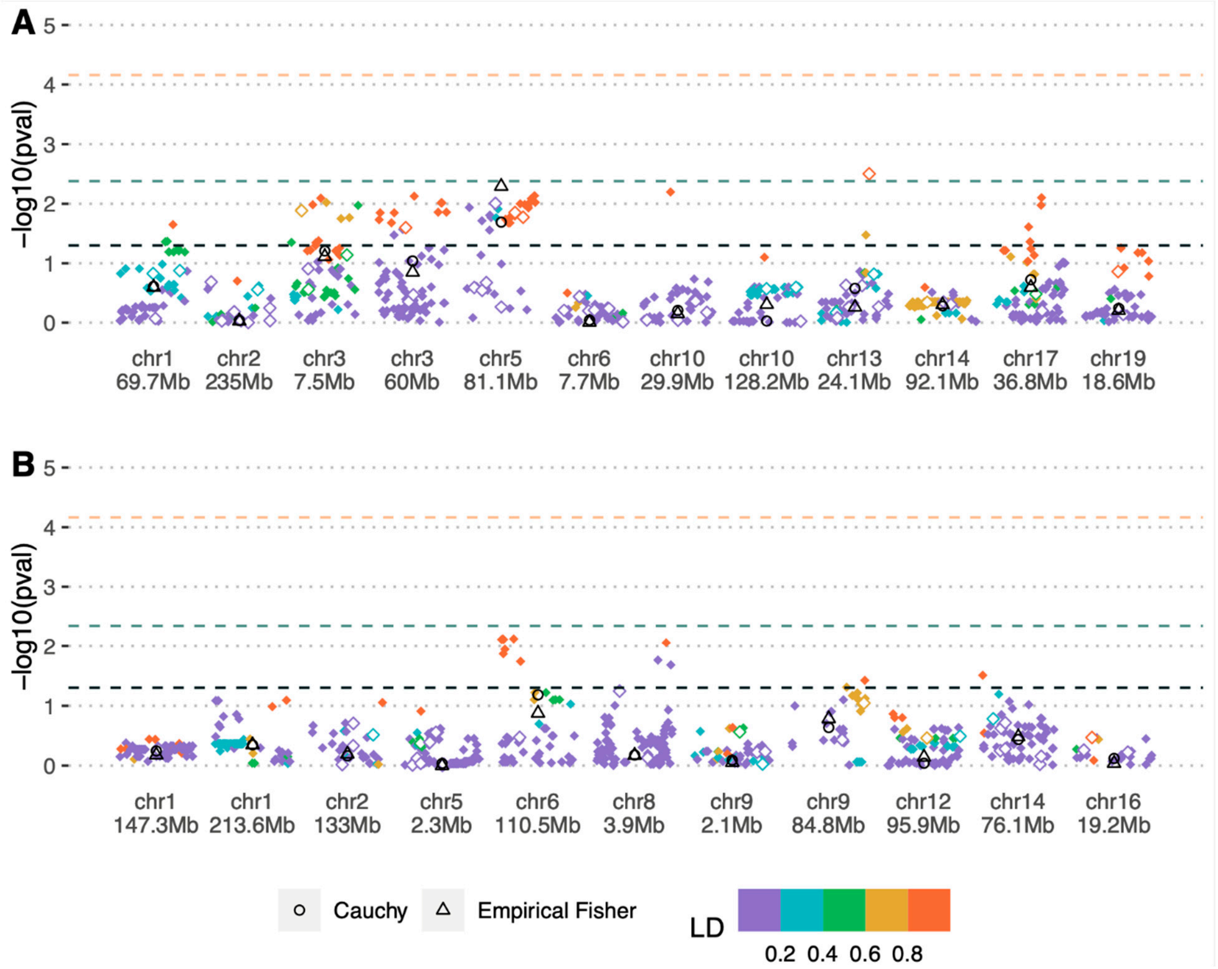

Supplementary Figure S3. Plots of the (A) maternal genetic effects and (B) parent-of origin effects. Colors represent linkage disequilibrium (LD) structure in the region measured by r^2 with the most significant SNP. SNP P-values are shown as diamond shapes, where filled shapes indicated imputed SNPs and open shapes indicate genotyped SNPs. Pooled empirical Fisher and Cauchy P-values are shown as open black triangles and circles. The black, green and orange dashed line indicate, respectively, the 0.05 significance level, the significance level for the pooled P-value Bonferroni-corrected for the number of regions tested, and the significance level Bonferroni-corrected for the total number of SNPs tested across all regions.

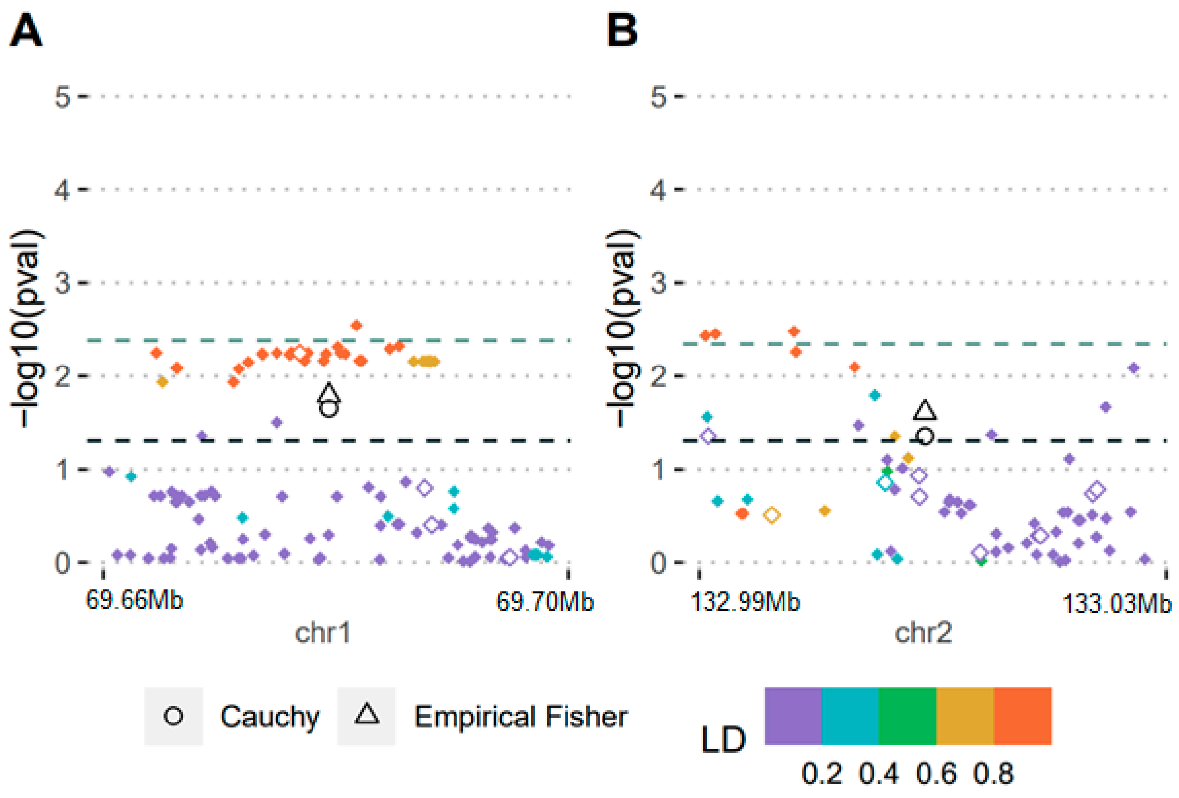

Supplementary Figure S4. Plots of the Wald interaction test for the extended regions for nominally significant region (A) on chromosome 1 (chr1 69.7Mb) for maternal genetic effects and folic acid supplementation, and (B) on chromosome 2 (chr2 133Mb) for parent-of-origin effects and smoking. Colors represent linkage disequilibrium (LD) structure in the region measured by r^2 with the most significant SNP. SNP P-values are shown as diamond shapes, where filled shapes indicated imputed SNPs and open shapes indicate genotyped SNPs. Pooled empirical Fisher and Cauchy P-values are shown as open black triangles and circles. The black and green dashed line indicate, respectively, the 0.05 significance level and the significance level for the pooled P-value Bonferroni-corrected for the number of regions tested.

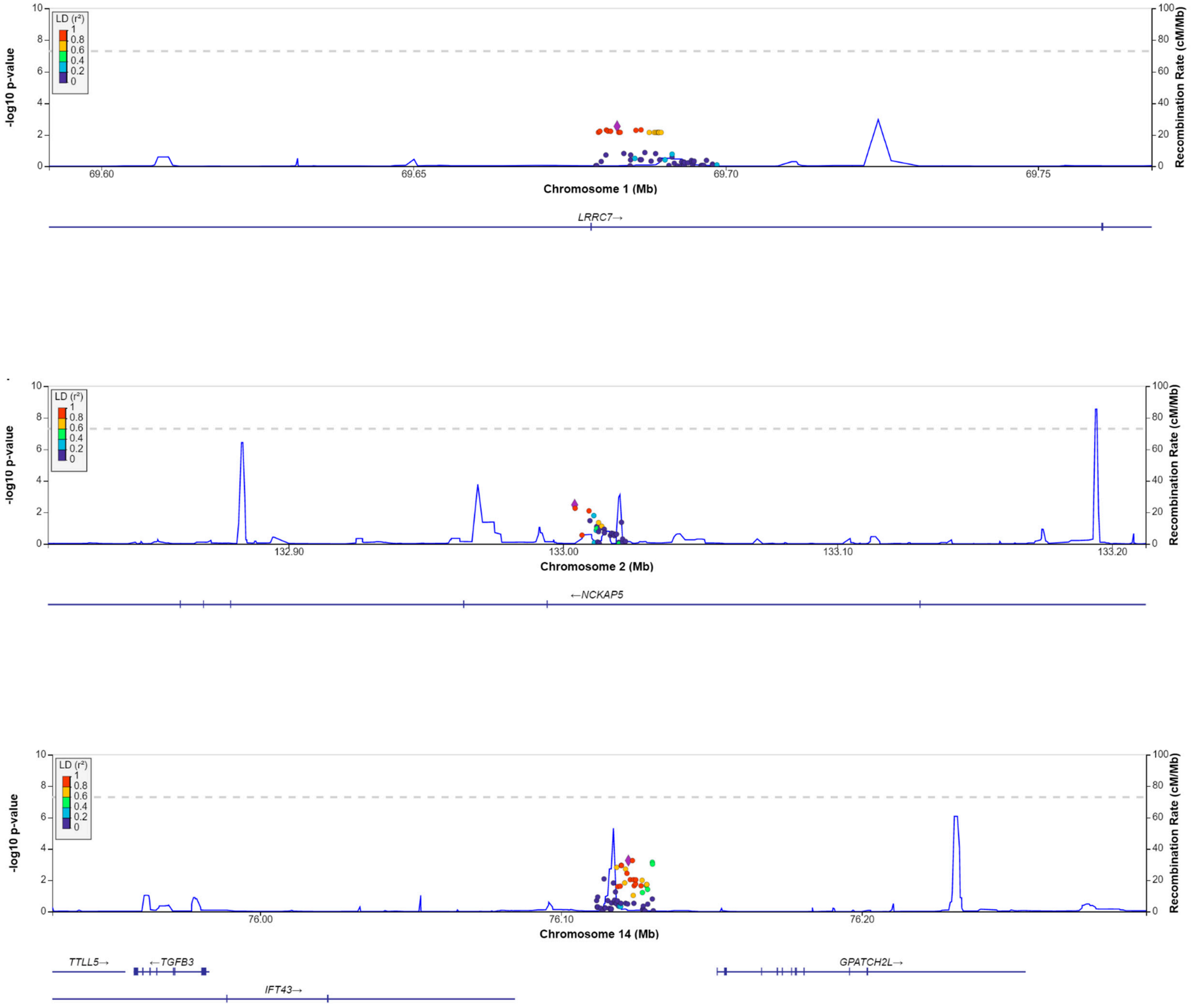

Supplementary Figure S5. LocusZoom plots showing recombination rates, linkage disequilibrium and nearby genes for the three nominally significant regions.

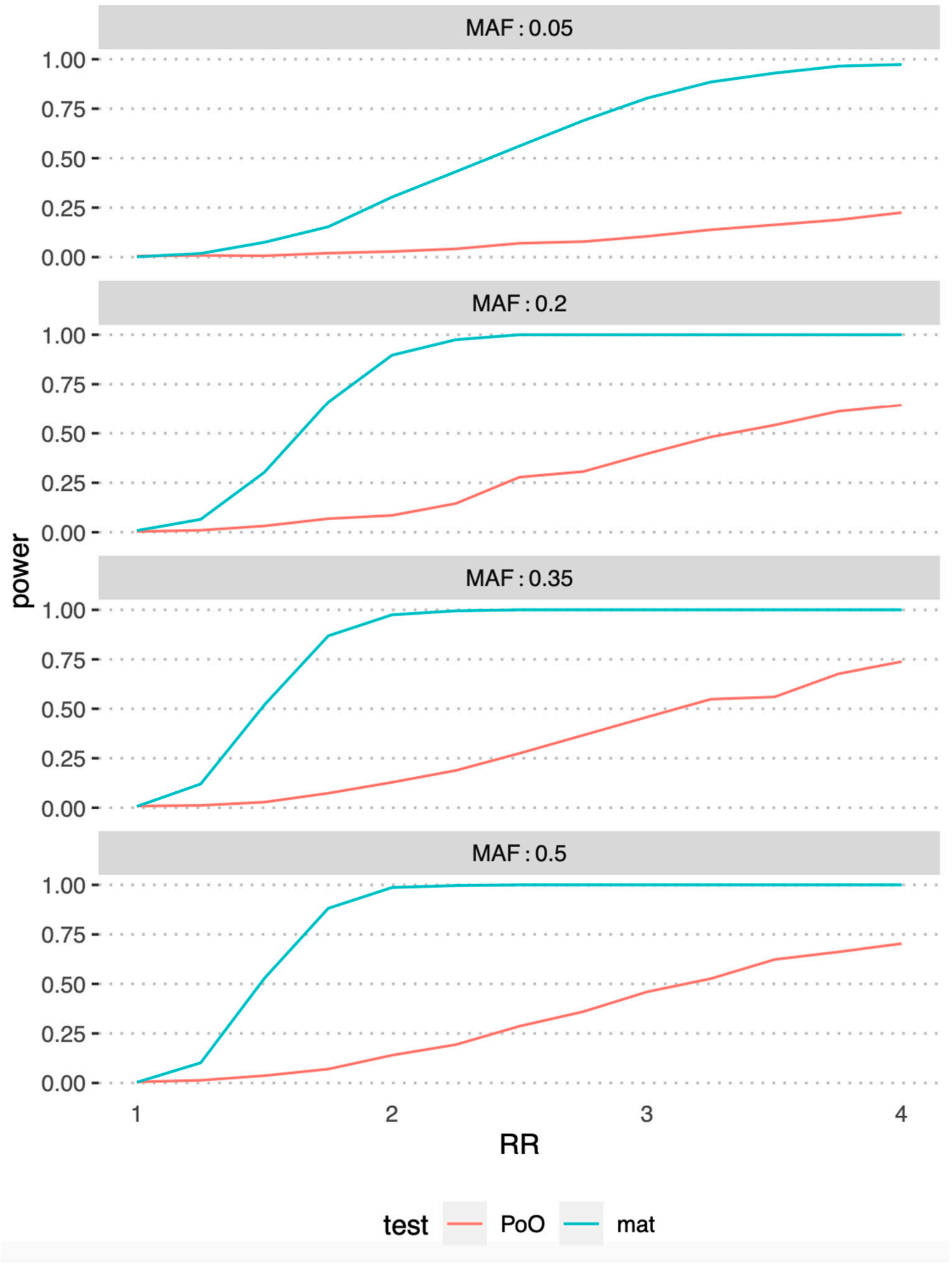

Supplementary Figure S6. Estimated power to detect the maternal (mat) and PoO interaction effects. We used the hapPower function of Haplin to estimate power for a range of interaction effect sizes comparable to those that we expect given previous literature. hapPower uses simulations to estimate power for the maternal- or PoO-environment interactions. We used the same number of triads/dyads in the simulations as in our sample. MAF: minor allele frequency of the lead SNP.
